# Vision loss after accidental methanol intoxication: a case report

**DOI:** 10.1186/1756-0500-6-479

**Published:** 2013-11-20

**Authors:** Marilita M Moschos, Nikolaos S Gouliopoulos, Alexandros Rouvas, Ioannis Ladas

**Affiliations:** 1 1st Department of Ophthalmology, University of Athens Medical School, ‘G. Genimmatas’ General Hospital Athens, 154 Mesogion Avenue, Holargos 11527, Athens, Greece; 2 2nd Department of Ophthalmology, University of Athens Medical School, ‘Attikon’ Hospital, 1 Rimini Street, Haidari 12462, Athens, Greece

**Keywords:** Methanol Intoxication, Bilateral Blindness, ERG, VEPs, Multifocal VEPs, OCT

## Abstract

**Background:**

Methanol intoxication is a dangerous situation because it often results in permanent problems such as visual deterioration, metabolic disturbances, neurological dysfunction, and even death. We present, to the best of our knowledge, the first case of irreversible bilateral blindness due to methanol intoxication caused by accidental ingestion of rubbing liquid.

**Case presentation:**

A 49-year-old Greek man developed bilateral irreversible blindness after accidental methanol intoxication. He underwent complete ophthalmological examination, including electroretinogram, visual evoked potentials, multifocal-visual evoked potentials, and optical coherence tomography scan of the optic nerve. Complete laboratory evaluation, urine drug testing, neurological examination, and computed tomography scans were also performed. Visual acuity demonstrated no light perception bilaterally, pupils were semi-dilated and unreactive to light, while the retina was normal in both eyes. Electroretinogram was normal, while visual evoked potentials, multifocal-visual evoked potentials recording, and optical coherence tomography scanning of both optic nerve heads were pathological in both eyes. The neurological examination and the computed tomography scans did not reveal any abnormalities. The laboratory evaluation was normal and the urine drug test was negative for benzodiazepines, opiates, cocaine, amphetamines, salicylates, barbiturates, and phencyclidine.

**Conclusion:**

This is the first case report of methanol intoxication which documents both anatomical and functional abnormalities by means of optical coherence tomography and electrophysiological tests correspondingly. The ocular findings and the reported electrophysiological changes support the hypothesis that methanol affects photoreceptors, Müller cells, and the retrolaminar portion of the optic nerve.

## Background

Methanol is a clear, colourless and volatile fluid which smells and tastes like ethanol and therefore it is difficult to differentiate methanol from alcohol. Its main uses are as a raw material for industrial solvents, antifreeze production and in bootleg whiskey. Accidental ingestion of methanol is common (more than half of the methanol intoxication cases), preventable and causes serious side effects. Methanol intoxication (even in small amounts) is very dangerous because it can cause severe visual dysfunction (including irreversible bilateral blindness), metabolic disturbances, permanent neurological dysfunction and even death [1]. We present, to the best of our knowledge, the first case of methanol intoxication caused by accidental ingestion of rubbing liquid.

## Case presentation

**Figure 1:**
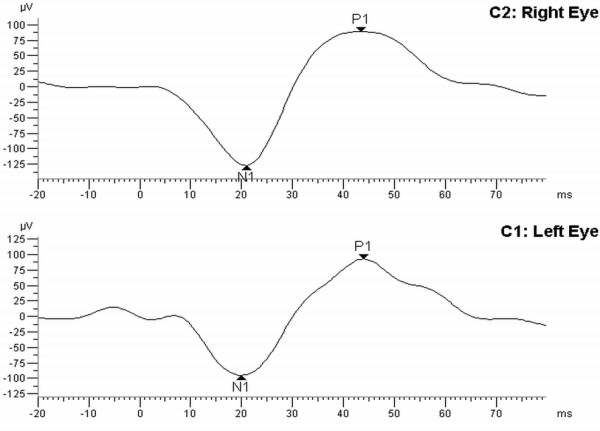
Electroretinogram recording; Electroretinogram recording is normal bilaterally.

**Figure 2:**
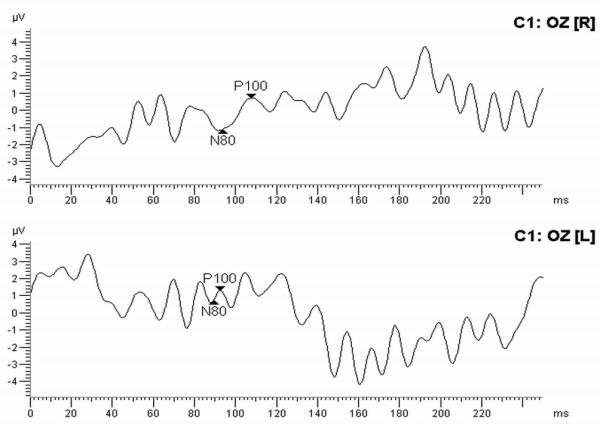
Visual evoked potential recording; Visual evoked potential recording is extremely decreased bilaterally.

**Figure 3:**
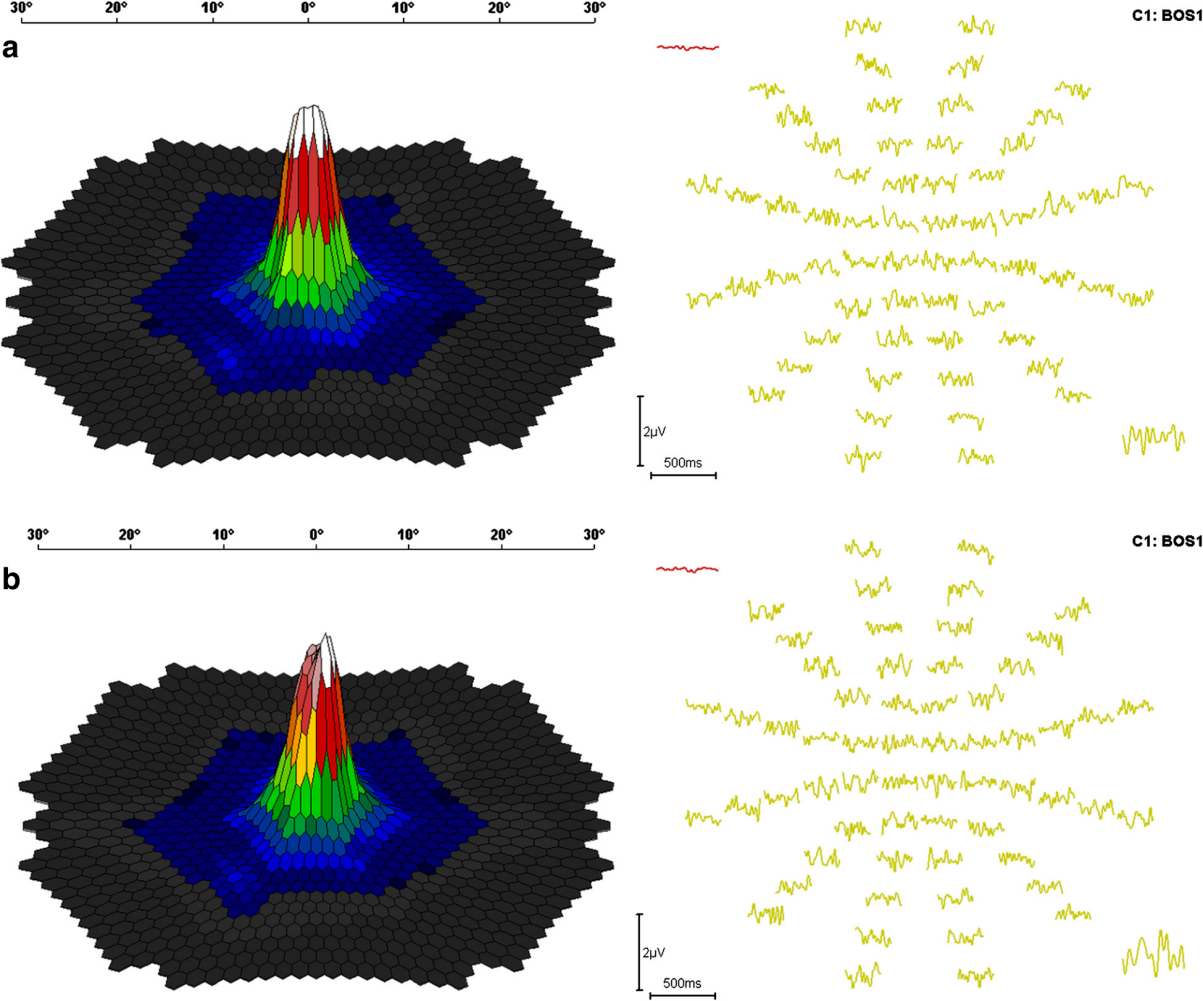
**Multifocal visual evoked potential recording; Multifocal Visual evoked potential 3D plot and corresponding traces of the right eye (3a) and the left eye (3b) respectively.** Area 0 is very diminished bilaterally (right eye: 169 nV/deg^2^, left eye: 186 nv/deg^2^).

**Figure 4:**
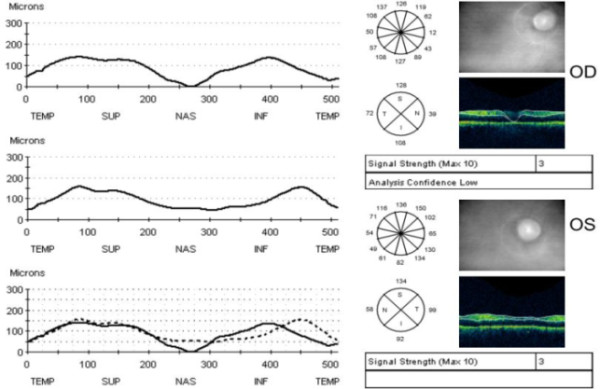
Optical coherence tomography recording around the optic nerve head; retinal nerve fiber layer thickness in the right eye (OD) in μm (128 in the superior, 39 in the nasal, 108 in the inferior, and 72 in the temporal quadrant) and retinal nerve fiber layer thickness in the left eye (OS) (134, 99, 92, and 58 correspondingly).

A 49-year-old healthy Greek man without any prior significant medical history, working as a cook on a merchant ship, drank accidentally a glass of 70% methanol rubbing solution, while he was on board. One day later he complained for blurred vision and painful eye movement in both eyes. The second day he woke up blind. He remained on board since the ship was heading to Australia and so treatment was impossible. When the ship reached Australia, he was hospitalized for a week. On arrival his vital signs were within normal limits and his examination tests revealed normal muscle tone. His initial laboratory evaluation included a complete blood count, electrolytes, blood urea nitrogen, creatinine, and serum glucose. All test results were within the normal range for the patient’s age. A urine drug test was negative for benzodiazepines, opiates, cocaine, amphetamines, phencyclidine, salicylates, and barbiturates. The blood methyl alcohol and formic acid values could not be determined. No treatment was given due to patient’s late arrival. He then was transferred to the University Eye Clinic of Athens. At presentation he underwent a complete ophthalmological examination. Visual acuity was no light perception in both eyes. The pupils were semi-dilated and unreactive to light. Fundus examination revealed an unremarkable retina in both eyes with the exception of pronounced pale, atrophic optic discs with “pseudoglaucomatous” thinning of the neuroretinal rim area. Electroretinogram (ERG) was normal in both eyes (Figure 1). Visual evoked potentials (VEPs) were nearly extinguished (Figure 2). Multifocal-visual evoked potential (mf-VEP) recording was also pathological in area 0 (right eye: 169 nV/deg2 and left eye: 186 nV/deg2) (Figure 3). Optical coherence tomography (OCT) of the optic nerve head demonstrated abnormally low values of the retinal nerve fiber layer (RNFL) thickness equal to 128 μm in the superior, 39 in the nasal, 108 in the inferior, and 72 in the temporal quadrant of the right eye (OD), and 134, 99, 92, and 58 correspondingly of the left eye (OS) (Figure 4).

Neurological examination with the patient awake revealed no extrapyramidal motor disturbances and computed tomography (CT) scans showed no abnormalities. Anion gap was less than 30 mg/dL and no treatment was deemed necessary to initiate. The patient was discharged on the fourth day. He was reexamined one month later. The situation remained unchanged.

## Discussion

Methanol intoxication is followed by a 12–24 hour latency period, after which appear its symptoms and signs which are related to the gastrointestinal, to the ocular, and to the nervous system. Early ocular findings include photophobia, blurred vision, painful eye movements, disturbances of pupil reactions and colour vision, decreased visual acuity, visual field defects and optic disc oedema with tortuous retinal vessels. Their absence on the initial examination is a good prognostic sign and there is not any case reported with permanent visual loss after an initial normal examination [2]. Myelin damage at the retrolaminar optic nerve has been shown in histopathological examination.

In our case, VEPs were almost extinguished and OCT scan of the optic nerve revealed highly decreased values. These findings are in accordance with the literature and are probably a result of the fact that methanol affects photoreceptors, Müller cells, and the retrolaminar portion of the optic nerve [3].

Methanol intoxication should be early recognised and treated in order to prevent permanent health problems and patient’s death. The treatment includes the treatment of the metabolic acidosis by oral or intravenous bicarbonate administration, the prevention of further oxidation of methanol to toxic metabolites (formaldehyde and formic acid) by competitive inhibition of the enzyme alcohol dehydrogenase (by ethyl alcohol or fomepizole), and the elimination of methanol and its metabolites by haemodialysis.

Our patient developed irreversible blindness bilaterally because he was not treated in the maximum time of two days since the methanol ingestion.

## Conclusion

This is the first case report of methanol intoxication which documents both anatomical and functional ocular abnormalities by means of OCT and electrophysiological tests correspondingly. The ocular findings and the reported electrophysiological changes support the hypothesis that methanol affects photoreceptors, Müller cells, and the retrolaminar portion of the optic nerve. It describes a case which has a broader clinical impact across medicine. Our case also underlines the importance of the early recognition and treatment initiation in cases of methanol ingestion.

## Consent

Written informed consent was obtained from the patient for publication of this case report and any accompanying images. A copy of the written consent is available for review by the Editor-in-Chief of this journal.

## Competing interests

The authors declare that they have no competing interests.

## Authors’ contributions

MMM participated in writing the case report and examined the patient. NSG participated in writing the case report. AR initially examined the patient. IL performed the OCT examination. All authors read and approved the final manuscript.
